# Status of national policy on companion of choice at birth in Latin America and the Caribbean: Gaps and challenges

**DOI:** 10.26633/RPSP.2020.19

**Published:** 2020-03-01

**Authors:** Bremen De Mucio, Lorena Binfa, Jovita Ortiz, Anayda Portela

**Affiliations:** 1 Latin American Center for Perinatology, Women and Reproductive Health Pan American Health Organization/World Health Organization Montevideo Uruguay Latin American Center for Perinatology, Women and Reproductive Health, Pan American Health Organization/World Health Organization, Montevideo, Uruguay.; 2 Departamento de Promoción de la Salud de la Mujer y el Recién Nacido, Facultad de Medicina Universidad de Chile Santiago Chile Departamento de Promoción de la Salud de la Mujer y el Recién Nacido, Facultad de Medicina, Universidad de Chile, Santiago, Chile.; 3 Department of Maternal, Newborn, Child and Adolescent Health and Ageing World Health Organization Geneva Switzerland Department of Maternal, Newborn, Child and Adolescent Health and Ageing, World Health Organization, Geneva, Switzerland.

**Keywords:** Patient satisfaction, midwifery, maternal health services, Latin America, Caribbean Region, Stisfacción del paciente, partería, servicios de salud maternal, América Latina, Región del Caribe, Satisfação do paciente, tocologia, serviços de saúde maternal, América Latina, Região do Caribe

## Abstract

The World Health Organization (WHO) recommends a companion of choice during labor and birth, to improve maternal and perinatal outcomes and women’s satisfaction with health services. To better understand the status of companion of choice in Latin America and the Caribbean (LAC), an online survey was conducted with members of a midwifery virtual community of practice and with key informants, aiming to identify: 1) existing regulatory instruments related to companion of choice in the countries where the members are practicing; and, 2) key characteristics of implementation of companion of choice, where regulation exists. Responses (*n* = 112) were received from representatives of 20 of the 43 countries of LAC. Respondents reported existence of a national policy or legislation in seven countries, ministerial norms or institutional protocols in five countries, and no existing policy/protocol in eight countries. Respondents from the same country often provided contradictory responses. Responses differed from information provided by ministries of health in a WHO-led global policy survey in 11 instances. These variations may reflect that midwives were not always aware of the national policy/guideline in their country. We propose that a more robust effort should be undertaken to understand the status of companion of choice for labor and birth in LAC countries, at national, regional, and local level, in public and private facilities. It is important to know if policies exist, at what level of the system, and if key stakeholders, maternity-care health providers, and women are aware of their existence. Efforts should also be made to understand barriers to implementing companion of choice.

## RESUMEN

La Organización Mundial de la Salud (OMS) recomienda la presencia de un acompañante durante el trabajo de parto y el parto debido a que mejora los resultados maternos y perinatales y la satisfacción de las mujeres con los servicios de salud. Para comprender mejor la situación acerca de los acompañantes en América Latina y el Caribe (ALC) se llevó a cabo una encuesta en línea dirigida a miembros de una comunidad de práctica de partería e informantes clave con el objetivo de identificar: 1) los instrumentos regulatorios existentes relacionados con la presencia de acompañante en los países en los que ejercen las personas encuestadas y 2) las características clave relacionadas con la implementación del acompañante, en los lugares donde existe un marco regulatorio. Se recibieron 112 respuestas de 20 de los 43 países de ALC. Las personas encuestadas informaron la existencia de una política o legislación nacional en siete países, de normas ministeriales o protocolos institucionales en cinco países, y de la inexistencia de una política o un protocolo en ocho países. Las respuestas provenientes del mismo país a menudo fueron contradictorias, y en 11 casos estas difirieron de la información proporcionada por los ministerios de salud en una encuesta mundial sobre políticas dirigida por la OMS. Estas variaciones pueden reflejar que los profesionales de la partería no siempre conocían la política o el protocolo de su país. Debe emprenderse un esfuerzo más firme para comprender la situación relacionada con el acompañante durante el trabajo de parto y el parto en los países de ALC a nivel nacional, regional y local, tanto en instituciones públicas como privadas. Es importante conocer si existen políticas y en qué nivel del sistema y si los principales interesados, los prestadores de servicios de salud materna y las mujeres conocen su existencia. Se deben realizar esfuerzos para comprender los obstáculos que impiden la implementación de la presencia de un acompañante durante el parto.

## RESUMO

A Organização Mundial da Saúde (OMS) recomenda a presença de um acompanhante durante o trabalho de parto e parto, já que essa medida melhora os resultados maternos e perinatais e a satisfação da mulher com os serviços de saúde. Para caracterizar a situação dos acompanhantes na América Latina e Caribe (ALC), realizou-se uma pesquisa ***on-line*** com membros de uma comunidade de prática de profissionais de obstetrícia e com informantes chaves para identificar: 1) a existência de instrumentos regulatórios relacionados com a presença de acompanhante nos países onde os respondentes atuam e 2) características chaves relacionadas com a implementação das políticas de acompanhantes nos locais onde existe regulamentação. Foram recebidas 112 respostas de 20 dos 43 países da ALC. Os respondentes relataram a existência de uma política ou legislação nacional em sete países, normas ministeriais ou protocolos institucionais em cinco países e nenhuma política ou protocolo em oito países. Respondentes de um mesmo país deram muitas vezes respostas contraditórias. Em 11 casos, as respostas diferiram das informações fornecidas pelos ministérios da saúde em uma pesquisa de políticas globais realizada pela OMS. Essas variações podem indicar que os profissionais nem sempre conheciam a política ou protocolo em vigor no seu país. Propõe-se a necessidade de iniciativas mais robustas para compreender a situação do acompanhante no trabalho de parto em países da ALC, em nível nacional, regional e local, tanto em instituições públicas como privadas. É importante saber se as políticas existem, em que nível do sistema existem e se as principais partes interessadas, os provedores de cuidados de saúde materna e as mulheres estão cientes de sua existência. São necessários esforços para compreender os obstáculos à implementação do sistema de acompanhante de parto.

WHO Recommendations: Intrapartum Care for a Positive Childbirth Experience (1) from 2018 include a recommendation for a companion of choice for all women throughout labor and childbirth. The companion can be any person the woman chooses to provide her with continuous support during labor and childbirth, including her husband/partner, a friend or family member, a community health worker, a traditional birth attendant, or a doula (i.e., a woman who has been trained in labor support but is not part of the health care facility’s professional staff) (1). This recommendation is based on evidence from a Cochrane systematic review that included 26 trials with data on 15 858 women from 17 countries (2). The review found that, compared with usual practice, companionship during labor and childbirth was likely to increase spontaneous vaginal birth, and decrease cesarean section, use of any type of pain relief, and negative feelings about the childbirth experience. The newborns of women who received continuous support may be less likely to have low five-minute Apgar scores, indicating possible benefits for infants as well.

The evidence on a companion of choice during labor and birth has been available for many years and is endorsed by key international associations including the International Federation of Gynecology and Obstetrics, International Confederation of Midwives, International Pediatric Association, and the White Ribbon Alliance (3, 4). Nonetheless, the practice often faces resistance from policy-makers and service providers (5).

Qualitative research confirms that women hope to have this continuous support to improve their childbirth experience (6). In a review of mistreatment during labor, Bohren et al. (2015) found that many women express the desire for support during childbirth, but that many facilities around the globe deny women the right to have a companion of choice present (7). In the review, the denial of a companion was an official facility policy in some study sites, highlighting an institutionalized inconsistency with the current evidence and practice recommendations.

## CURRENT CHALLENGES AND GAPS FOR LATIN AMERICA AND THE CARIBBEAN

Little is known about the availability of companion of choice during labor and birth at health facilities in Latin America and the Caribbean (LAC), but it is believed to vary between and within countries. Some countries, including Argentina and Uruguay, have passed legislation, but research from Latin American countries on this topic is limited (8). Studies were conducted mainly in Brazil, and a few in Chile, where companion of choice is implemented. Women and their companions reported a positive experience, based on feelings of physical and emotional well-being, less fear and pain, and increased self-confidence (9–11). Health teams also reported a positive experience; however, reluctance to allow companionship was also reported (12–14).

The Pan American Health Organization (PAHO) is aligned with global and regional commitments, such as the 2030 Agenda for Sustainable Development and the Global Strategy for Women’s, Children’s and Adolescents’ Health, and their adaptation to the Region in the Sustainable Health Agenda for the Americas 2018–2030 (15–17). PAHO understands that companion of choice can contribute to fulfilling those international agreements, in particular the three objectives of the Global Strategy for Women’s, Children’s and Adolescents’ Health: Survive, Thrive, and Transform (16). Companion of choice could be a way to ensure the rights of women and their families, in providing respectful maternity care for women and newborns, reducing the number of interventions without medical justification, and improving the quality of care and the experience of care.

To better understand the policy situation, we conducted an online survey to identify: 1) existing regulatory instruments related to companion of choice in LAC countries; and, 2) key characteristics of implementation of companion of choice, where regulation exists.

## MATERIALS AND METHODS

An online survey to address the information gap was conducted from April to June 2015 using a brief questionnaire available in four languages (English, French, Portuguese, and Spanish) and compatible with different web browsers. The questionnaire was tested with selected faculty members of the University of Chile. It included closed-ended questions with multiple choice responses. Respondents were asked about the existence of regulatory instruments and the type of regulation (national policy, norms, decrees, institutional protocols) for companion of choice; as well as implementation components including practices to orient women, companions, and health providers; and the characteristics of companions. If the respondent indicated in the first question that there was no regulation in their country, the platform ended the questionnaire. An individual link allowed each respondent to complete the questionnaire only once.

The intended participants included midwifery providers in health services (clinicians), teaching universities, and management in public or private institutions. In a first round, an invitation to participate was e-mailed to members of a midwifery virtual community of practice, managed by the WHO Collaborating Centre for Development of Midwifery at the University of Chile. Given the limited number of initial responses, to solicit broader participation, an e-mail invitation was then sent to midwifery key informants in countries from which no responses had been received.

Participation in the survey was anonymous and voluntary; all questionnaire respondents were considered to have given their informed consent to participate. Ethical approval was not requested, as the survey did not record any personal information and was designed only to capture information regarding the existence of a potential public benefit. The survey was managed through the SurveyMonkey platform; data were collated in an Excel spreadsheet and analyzed using the Stata 13.0 statistical package.

## RESULTS

A total of 112 participants completed the survey, from 20 of the 43 LAC countries and territories (Table 1). Respondents were mostly teachers from public (34.7%) and private (10.2%) universities, clinicians from the public system (26.5%) and private sector (11.3%), and stakeholders from the public system (14.1%) and private sector (3.2%). The number of respondents per country varied: 39 from Chile; 10 from Mexico; 5–8 from Argentina, Brazil, Ecuador, El Salvador, Peru, and Uruguay; and 1–3 from 12 other countries (Table 1).

Among the 20 countries on which responses were received, 7 countries were reported to have a national policy or legislation; 5 to have ministerial norms or institutional protocols, and 8 to have no existing policy or legislation. Four countries were reported to have a national policy: Argentina, Brazil, Peru, and Uruguay.

For those nine countries where respondents indicated that an orientation was provided to the companion, two indicated that orientation was provided during pregnancy, three that it was provided upon admission to the facility for birth, and four that it was provided both during pregnancy and during admission. Only six countries reported that orientation or educational materials were available for health staff (Table 2).

Regarding the characteristics of companions of choice permitted, the majority reported the husband was allowed, followed by a relative or friend. The responses of two countries did not indicate that the husband was allowed; however, the survey did not capture if there were restrictions on men being present. Nine countries were reported to allow continuous support, meaning that the companion could remain throughout labor and birth; in three countries the companion was only allowed during labor; and in four the companion was allowed during cesarean section as well as vaginal births (Table 2).

**TABLE 1.** Survey responses on existence of regulations for companion at birth, by country

(A) Country	(B) Respondents (*n* = 112)	(C) Knowledge of existing regulation		(D) Existing national policy or guideline
		Yes	No	
Argentina	6	3	3	Yes
Barbados	1	1		(…)
Belize	1		1	Yes
Bolivia	3	1	2	Yes
Brazil	8	8		Yes
Chile	39	37	2	Yes
Colombia	1	1		Yes
Costa Rica	2	2		Yes
Dominican Republic	3		3	(…)
Ecuador	5	5		Yes
El Salvador	8		8	Yes
Guatemala	1		1	Yes
Haiti	1		1	Yes
Jamaica	2		2	NA^*^
Mexico	10		10	Yes
Paraguay	3	1	2	Yes
Peru	6	4	2	Yes
Puerto Rico	2	2		NA^*^
Trinidad and Tobago	2		2	Yes
Uruguay	8	8		Yes
Total	112	73	39	

* Did not participate in the survey (…) missing information ***Source:*** Columns A to C: prepared by authors from the survey results; Column D: from Global Reproductive, Maternal, Newborn, Child and Adolescent Health Policy Survey 2018–2019. Geneva: World Health Organization (unpublished).

## DISCUSSION

The WHO recommends a companion of choice during labor and birth for the improvement of maternal and perinatal outcomes and women’s satisfaction with health care services (1). Having a labor companion has been shown to impact positively on women’s experience of care (1, 11). Given its importance, we attempted to understand the prevalence of this practice in LAC countries. We reached out to midwifery personnel, assuming that they would be knowledgeable about this practice in their settings. The participants’ responses from 20 countries show that the situation regarding companion of choice in LAC varies in terms of the type and scope of legal instruments and institutional norms in place. Some respondents indicated that no formal legal or institutional instruments were available.

The fifth round of the Global Reproductive, Maternal, Newborn, Child and Adolescent Health Policy Survey was conducted in 2018–2019, using an online questionnaire administered to each WHO Member State. It included a question on policy related to companion of choice during labor and birth. Thirty countries or territories of the Americas participated in the Global Policy Survey and 22 indicated their country had a national policy or guideline that recommended the presence of a companion of choice during labor and birth. We compared the results of the Global Policy Survey with the responses obtained in the midwifery community of practice survey, and included these in Table 1. Comparison was possible for 16 of the 20 countries presented, and in 11 instances there was a difference between the responses in the Global Policy Survey and those from individual respondents in the midwifery community of practice survey. In two cases, the Dominican Republic and Mexico, the difference can be explained by the fact that legislation was introduced subsequent to the midwifery community of practice survey.

In addition, in the midwifery community of practice survey, in five cases, multiple respondents from the same country provided contradictory responses. This may be due to decentralized systems and regional variations within countries impacting on knowledge of national laws. However, the contradictory responses and the difference from the Global Policy Survey could indicate that midwifery personnel may not know about regulations and guidelines regarding companion of choice in their country.

If midwifery personnel are unaware, we speculate whether women are aware of the right to a companion. Two studies in Brazil showed that most childbearing women were not aware of their right (18, 19), despite the existence since 2005 of a national law supporting companion of choice. Furthermore, there are less than expected levels of implementation of this recommendation, which we attribute mainly to policy restrictions or inadequate provision in health facilities; however, we believe it could also reflect resistance from health staff to the participation of the companion (14, 20, 21), as well as a lack of commitment to empowering women to actively participate in their childbirth experience (7). The general perception of respondents is that a companion is more frequent during vaginal birth than in cesarean section, and continuous rather than partial, although findings from other studies show that companionship is frequently not continuous throughout labor and birth (20) and that companionship during labor is not always allowed (14).

**TABLE 2.** Survey responses on characteristics of regulation, by country (for those countries where respondents indicated a regulation exists)

Country	Type of regulations identified	Continuous or partial companionship allowed	Type of birth for which companion allowed	Companion of choice allowed	Orientation provided to health teams	Orientation provided to companions
Argentina	National policy MOH	Continuous ^a^	Vaginal	Husband	None	None
Barbados	Ordinance Decree	Continuous	Vaginal	Relative	(…)	(…)
Bolivia	Ordinance Decree/ IP	Continuous	CS	Friend	Orientation session(s)	Orientation upon admission at birth
Brazil	National policy MOH	Continuous	Vaginal	(…)	Orientation session(s)	Orientation provided during pregnancy; Orientation upon admission at birth
Chile	MOH IP	Continuous	Vaginal	Relative	None	Orientation provided during pregnancy; Orientation upon admission at birth
Colombia	MOH	Partial ^b^	CS	Friend	Educational materials	Educational material during admission
Costa Rica	MOH/IP	Partial	Vaginal	Husband	Orientation session(s) Educational materials	Orientation provided during pregnancy Educational materials provided upon admission at birth
Ecuador	IP	Partial	CS	Relative	(…)	Orientation during pregnancy
Paraguay	IP	Continuous	Vaginal	Friend	(…)	(…)
Peru	National policy MOH IP	Continuous	Vaginal	Husband	Orientation session(s)	Orientation upon admission at birth
Puerto Rico	Ordinance Decree IP	Continuous	Vaginal	Relative	Orientation session(s)	Orientation provided during pregnancy; Orientation upon admission at birth
Uruguay	National policy MOH	Continuous	(…)	Friend	None	Orientation during pregnancy

***Source:*** prepared by authors from the results.

MOH, Ministry of Health; IP, Institutional protocols; CS, Cesarean section

a During labor and birth

b During labor or birth

(…) missing information

Orientation of the woman, her companion, and health providers is considered important to ensure the quality of the intervention and that women, companions and health providers can work together as a team. Responses showed that this orientation is generally offered, with one country indicating it is not offered, and two with no response.

This exercise had methodological limitations, including a convenience sample. The number of responses per country varied widely, from 1 to 39. Nor can we generalize to all LAC countries, as less than a half of the countries in the region were represented. Participation was voluntary; those who participated were not selected based on their knowledge of national policy and legislation, and we made an assumption that midwives would be aware of policies on companion of choice. As mentioned, for five countries there were variations among participants in responses to some of the questions.

Despite these limitations, this exercise raises questions regarding midwives’ knowledge of legislation in their country and confirms that more robust efforts are needed to clarify not only the policy status on companion of choice during labor and birth but also key implementation characteristics, and to ensure its availability to all women in LAC.

## CONCLUSIONS

Although the evidence on companion of choice during labor and birth has been available for many years and is endorsed by key international associations, it appears that there are countries in LAC which have not implemented companion of choice, or that any existing laws are not yet well known. A more robust effort should be undertaken to understand the status of companion of choice for labor and birth in LAC countries at national, regional, and local level, in public and private facilities. It is important to know if policies exist, at what level of the system, and if key stakeholders including maternity-care health providers and women are aware of them. Efforts to understand barriers to implementing this important recommendation are also needed. Monitoring frameworks that capture implementation will help improve quality of care and ensure women and newborns receive the quality of care they are entitled to. WHO and PAHO call upon key partners including governments, United Nations sister agencies, professional organizations, international organizations, and civil society organizations to work together to advance this important recommendation to improve the well-being of women and newborns.

### Author contributions.

BDM and LB conceived the study and designed the instrument. LB and JO led the data collection and analysis. LB and AP interpreted the results and wrote the paper. All authors reviewed and approved the revised final version.

### Acknowledgments.

Our sincere thanks to the participants who responded to the survey; and to Theresa Diaz and Elizabeth Katwan from WHO Department of Maternal, Newborn, Child, and Adolescent Health and Ageing for their support in providing the data we used from the SRMNCAH Policy Survey.

### Disclaimer.

Authors hold sole responsibility for the views expressed in the manuscript, which may not necessarily reflect the opinion or policy of the *RPSP/PAJPH* and/or PAHO.
